# A prickly situation: an attempted Caterpillar ingestion - case report

**DOI:** 10.1186/s40463-020-00470-1

**Published:** 2020-09-29

**Authors:** Amar K. Bhardwaj, Naif Fnais, Christopher J. Chin

**Affiliations:** 1 Dalhousie Medicine New Brunswick, Saint John, New Brunswick Canada; 2 Department of Otolaryngology- Head and Neck Surgery, McGill University, Montreal, Quebec, Canada; 3 Department of Otolaryngology- Head and Neck Surgery, King Saud University, Riyadh, Saudi Arabia; 4 Department of Surgery, Division of Otolaryngology – Head and Neck Surgery, Saint John, New Brunswick Canada; 5 Department of Surgery, Division of Otolaryngology – Head and Neck Surgery, Dalhousie University, Halifax, Nova Scotia Canada

**Keywords:** Foreign body ingestion, Caterpillar, Otolaryngology, Case report

## Abstract

**Background:**

Foreign body ingestion is a common problem in pediatrics. Each foreign body can present its’ own unique challenges during removal, and we present the management of an ingested Spotted Tussock Moth (*Lophocampa maculata*), more commonly known as a caterpillar.

**Case presentation:**

An 18-month-old boy presented to the emergency department with difficulty handling secretions and odynophagia. It was reported he had placed a caterpillar in his mouth and then spat it out. On examination, hundreds of miniscule filaments (setae) were seen embedded in his lips and tongue. Our service was consulted out of concern for airway involvement. The patient was taken to the operating room where a direct laryngoscopy under general anesthesia with spontaneous ventilation was performed to confirm the setae were confined to the anterior tongue and lips. Once we were satisfied the airway was stable, the airway was secured, and we then began to remove the setae. The initial method used was to use Adson-Brown forceps to remove the setae, however this proved difficult and time-consuming given the volume of setae and how thin the setae were. Ultimately, a more effective technique was developed: a 4 × 4 AMD-RITMES® gauze was applied to the mucosa in order to dry up any secretions and then a piece of pink, waterproof BSN medical® tape was applied to the mucosa. After 3 s of contact it was removed. This technique was then repeated and was used to remove the vast majority of the setae.

**Conclusion:**

To our knowledge, we have described the first technique to remove the caterpillar setae from the oral cavity mucosa in a fast, safe and efficient manner.

## Background

Foreign body (FB) ingestion is a common problem in pediatrics and can present with upper airway obstruction. The presentation is variable depending on the FB, and whether the FB is aspirated on ingestion, but it can present with drooling, choking, dysphagia, odynophagia and chest pain. We present an unusual case of a (fortunately) failed attempt to ingest a caterpillar in an 18-month-old boy. The case highlights the difficulty in treating each unique FB, as the caterpillar ‘spines’ presented a challenge for removal.

## Case presentation

**Fig. 1:**
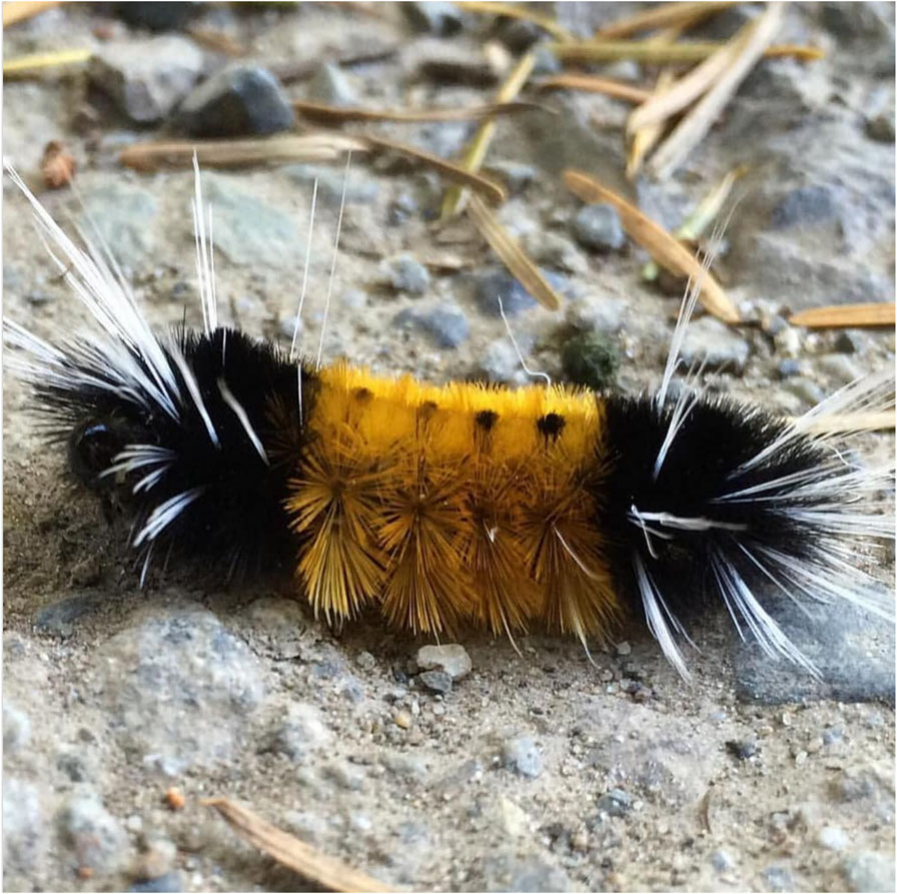
Image of the Spotted tussock moth Caterpillar (*Lophocampa maculata*) [1]

An 18-month-old boy presented to the emergency department with difficulty handling secretions and odynophagia. It was reported by his parents he had placed a caterpillar in his mouth and then spat it out. The caterpillar was identified as a *Lophocampa maculata* (Spotted tussock moth) by visual comparison to other referenced images by the parents (Fig. 1). On examination, hundreds of miniscule filaments (setae) were seen embedded in his lips and tongue. The patient was assessed in the Emergency department and, because of the difficulty handling secretions, Otolaryngology- Head and Neck Surgery was consulted.

**Fig. 2:**
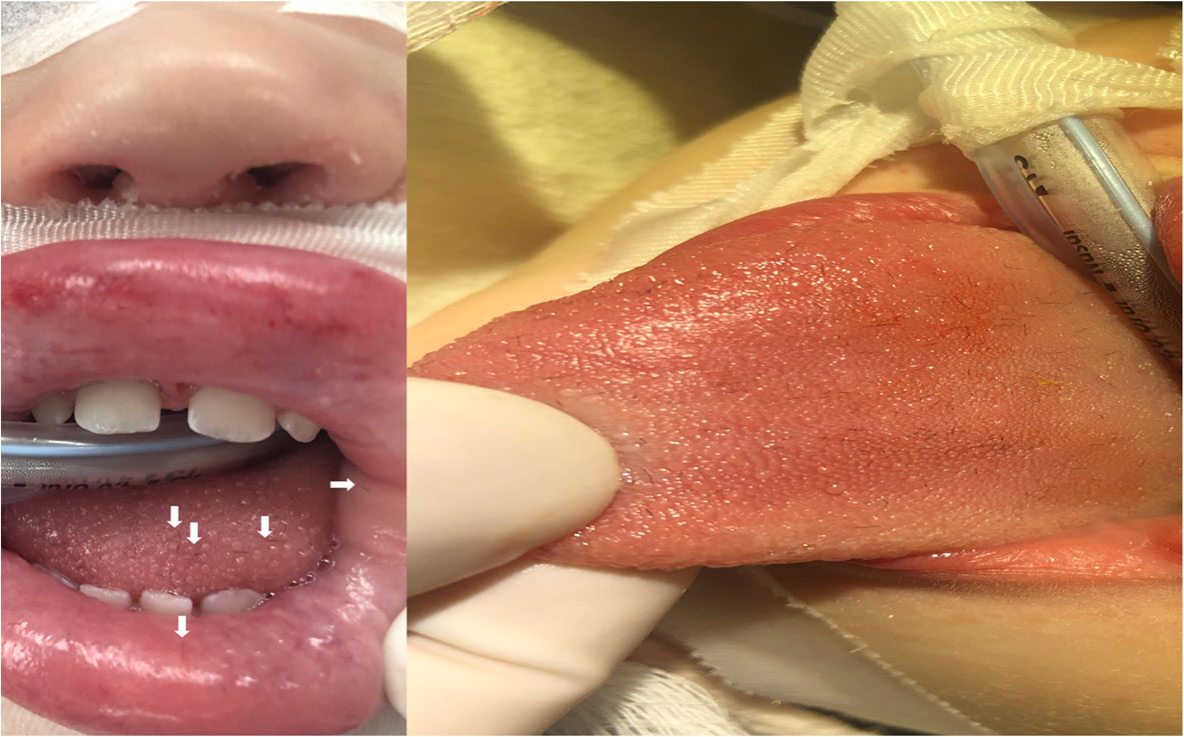
Miniscule filaments (Setae) embedded (arrowheads) in the tongue and lips

Upon initial assessment, the patient was drooling significantly but there was no stridor or dysphonia heard. The anterior tongue, buccal mucosa and lips were coated in setae (Fig. 2). It was decided the patient should be examined in the operating room to rule our laryngeal/hypopharyngeal involvement, and to remove the setae. The patient was taken to the operating room. After induction of general anaesthesia with spontaneous ventilation, direct laryngoscopy was performed to assess the upper airway and oropharynx. There was no evidence of setae in the oropharynx or larynx and it was confirmed the setae were confined to the anterior tongue and lips, with no evidence of upper airway edema. Once we were satisfied the airway was stable, the airway was handed back to the anesthesia service and secured via endotracheal intubation.

At this point, we began the removal of the setae from the patient’s lip, tongue and buccal mucosa. The initial method used was to use Adson-Brown forceps to remove the setae, however this proved difficult and time-consuming given the large volume and how fine the setae were. Ultimately, a more effective technique was developed: a 4 × 4 AMD-RITMES® gauze was applied to the mucosa in order to dry up any secretions and then a piece of pink, adhesive waterproof BSN medical® tape was applied to the mucosa. After approximately 3 s of contact, the tape was removed. This technique was then repeated and was used to remove the majority of the setae. It should be noted that not all small filaments were removed, with a few left embedded in the mucosa and within the tongue papillae, as these were quite difficult to extract. The patient was then reversed from general anesthesia, extubated and transferred to the post-anesthesia care unit in stable condition. The child was admitted overnight to observe for any potential complications such as delayed systemic toxicity. The patient was discharged the following day with no further issues.

## Discussion

Ingested foreign bodies are a common clinical problem primarily seen in children; 56.6% of patients are between 1 and 3 years of age [2]. The most common site for a foreign body to get stuck is the hypopharynx, particularly at the cricopharyngeal sphincter [2]. The foreign body presented in this case is a well-known insect: The Caterpillar, with over 150,000 different species described [3]. In Canada, namely, New Brunswick, there are 1593 described variants [3], some of which are poisonous. On appearance, caterpillars have spines and hair like structures on their exterior. In some species, it serves as a defence mechanism and the hairs (setae) and spikes can sting their foe, leading to adverse reactions in the recipient, mainly cutaneous reactions, include dermatitis and localized reactions.

Lepidopterism (the general term which refers to the toxic effects of caterpillars) leads to clinical signs that vary from urticarial wheels to conjunctivitis, pharyngitis, malaise, and rarely, anaphylactic reactions [4]. Therefore, minimizing exposure to the setae in the oral cavity and oropharynx is important in these cases. To our knowledge, this is the first reported case of oral cavity exposure to the *Lophocampa maculata* species, and we have reported on a method to remove the setae atraumatically in this location. While tape has been used anecdotally to remove the setae from the skin, in the oral cavity the saliva makes it considerably less effective, unless the mucosa is dried first with gauze.

There are toxic strains of caterpillars to be aware of, namely the Hickory tussock (*Lophocampa caryae*) and Io moth caterpillar (*Automeris io*). The Hickory tussock moth caterpillar has a characteristic row of black tufts that are responsible for the caterpillar’s toxicity along with white hairs along its side. Envenomization occurs when the recipient presses their hairs that release toxins such as proteolytic enzymes, histamine and other pro inflammatory substances [5]. The Io moth caterpillar (*Automeris io*) has a pale yellow to green color with red true legs and prolegs. These caterpillars release their venom from the tips breaking off when they penetrate skin leading to subsequent irritation. In our case, there was a risk of venom release with subsequent airway edema, and potentially, critical airway obstruction. Therefore, it was imperative to quickly evaluate and secure the airway. Balit et al. have described in a report pertaining to the cutaneous manifestations of the white-stemmed gum moth that attempted removal of all setae is near impossible, non-essential and time consuming [6]. Another case report describes an anaphylactic reaction to the *Lophocampa maculata* species with cutaneous manifestations [7]. The patient’s anaphylactic reaction manifested with an acute development of diffuse urticaria along with progressive dyspnea [7]. To our knowledge, this is the first reported case of oral cavity exposure to the *Lophocampa maculata* species, and we have reported on a method to remove the setae atraumatically in this location. While tape has been used anecdotally to remove the setae from the skin, in the oral cavity the saliva makes it considerably less effective, unless the mucosa is dried first with gauze.

## Conclusion

Following potential ingestion of a caterpillar, the setae embedded in the oral mucosa should be removed in order to minimize potential complications. To our knowledge, we have described a novel technique to remove the caterpillar setae from the oral cavity mucosa in a fast, safe and efficient manner. The use of adhesive waterproof tape, preceded by application of gauze to dry up the mucosa to allow good contact, allowed for timely removal of caterpillar filaments.

## Data Availability

Not applicable.
